# Intraindividual Variability in Executive Function Performance in Healthy Adults: Cross-Sectional Analysis of the NAB Executive Functions Module

**DOI:** 10.3389/fpsyg.2018.00329

**Published:** 2018-03-14

**Authors:** Dorota Buczylowska, Franz Petermann

**Affiliations:** Center of Clinical Psychology and Rehabilitation, University of Bremen, Bremen, Germany

**Keywords:** executive functions, intraindividual variability, dispersion, cognitive aging, NAB

## Abstract

The current study was aimed at investigating across-tasks intraindividual variability, also termed dispersion, in EF performance. The German adaptation of the Neuropsychological Assessment Battery (NAB) was used as a measure of EFs. Data of 444 participants aged 18–99 from six NAB Executive Functions Module subtests (i.e., Planning, Mazes, Letter Fluency, Judgment, Categories, and Word Generation) along with the NAB Total Index score as a measure of overall cognitive ability were analyzed. Maximum discrepancy (MD) was applied as a measure of dispersion. MD values ranged from 0.47 to 5.20 indicating substantial across-tasks dispersion in EF performance. Furthermore, dispersion moderately decreased with advancing age. Taking overall cognitive ability into account revealed that dispersion might be lower at older ages; especially, when associated with low overall ability levels. The dedifferentiation hypothesis offers a plausible explanation for these findings. That is, the cognitive profiles of older people might be less heterogenous than that of younger people, which may be due to age-related central nervous system constraints.

## Introduction

There is a substantial degree of variability in the literature regarding the conceptualization and operationalization of executive functions (EFs). A recently conducted review of contemporary empirical studies, however, revealed some points of convergence among researchers in respect to the definition of EFs (Baggetta and Alexander, 2016). According to this review, the majority of researchers regards EFs as a set of cognitive processes responsible for guiding and monitoring action and behaviors crucial to learning and everyday human performance tasks. Furthermore, executive functioning is considered more a multidimensional construct comprising several cognitive processes rather than single ability. Nevertheless, no consensus among researchers has been reached in respect to which cognitive processes comprise this multidimensional construct (Maricle and Avirett, 2012; Flanagan et al., 2014). According to Baggetta and Alexander (2016), researchers identified 39 different components as EFs. Inhibitory control, working memory (WM), shifting or cognitive flexibility were among components or labels most frequently encountered in the literature. Planning and attention were other terms often used by researchers in relation to executive functioning. Even less consensus appears to be regarding the operationalization of the EF construct. Within the 106 studies reviewed by Baggetta and Alexander (2016), 109 different tasks, and 11 different batteries were used as measures of EFs. Moreover, the same tasks were used in different studies to measure different components or single tasks were used to measure multiple components. An important conclusion of the review is that researchers should work toward a unifying definition and develop new assessment tools to measure EFs.

When exploring the nature of EFs, developmental patterns are to be taken into account. In particular, evidence exists on age-related individual differences in EFs across the entire life span (De Luca et al., 2003; Romine and Reynolds, 2005; De Luca and Leventer, 2008; Reynolds and Horton, 2008; Buczylowska and Petermann, 2016). Particularly interesting are the findings supporting the differentiation-dedifferentiation hypothesis, which postulates that cognitive abilities change throughout the life span in the extent of relationship. That is, EFs in children are unidimensional, but develop through adolescence into early adulthood into a multidimensional construct (Wiebe et al., 2011; Brydges et al., 2012); with advancing age and reaching late adulthood, however, EFs become more unidimensional again (Buczylowska et al., 2016; Buczylowska and Petermann, 2017).

The existing evidence on developmental patterns in EFs has been gained using both indices of central tendency and measures of interindividual variability. Studying age-related differences between individuals is particularly important as the magnitude of heterogeneity in EFs may help better understand why some individuals display changes in EFs, whereas others do not (Buczylowska and Petermann, 2016). Nevertheless, it has been acknowledged that exploring within-person differences in cognitive performance, also termed intraindividual variability (IIV), may also provide important information in respect to developmental changes associated with normal and pathological aging.

It should be noted that the term IIV refers either to differences within individuals across different tasks within single occasion (dispersion) or differences within individuals within one task across multiple occasions (Hultsch et al., 2002; Hilborn et al., 2009; inconsistency). Studies have shown that both dispersion and inconsistency in cognitive performance might increase with advancing age (Christensen et al., 1999; Hultsch et al., 2002; Schretlen et al., 2003; Williams et al., 2005; Hilborn et al., 2009; Vandermorris et al., 2013; Ferreira et al., 2017). Nevertheless, contrary results have also been reported (Lindenberger and Baltes, 1997; Rapp et al., 2005; Mella et al., 2016). It has been argued that increases in IIV might be observed in younger and healthy adults, whereas decreases in IIV might follow very late in life as a result of age-related cognitive decline (Hultsch et al., 2002). However, this is normal aging that seems to be associated with decreases in IIV in late adulthood. Cerebral dysfunctions, in their initial stages especially, may present specific deficits in one cognitive domain, resulting in an increase of cross-domain variability (Rapp et al., 2005). This is consistent with the findings demonstrating links between increased IIV and lower cognitive functioning (Christensen et al., 1999; Rapp et al., 2005; Hilborn et al., 2009) as well as higher risk for dementia (Hultsch et al., 2000; Holtzer et al., 2008).

Advances in the study of IIV might have practical implication for clinical neuropsychology (Hilborn et al., 2009; Vandermorris and Tan, 2015). In particular, based on prior research, the characteristics of psychometric assessment must be reconsidered. As an example, fluctuations in test performance across different occasions may question the validity and reliability of the measures used. IIV in performance across different tasks on a single occasion also presents a challenge in respect to clinical decisions; that is, neuropsychological evaluations are based on comparisons of an individual with a norm of a similar developmental cohort. Consequently, if there is substantial dispersion within individuals in respect to test scores, better understanding of developmental profiles, and their potential associations with normal and pathological aging is required (Vandermorris and Tan, 2015).

Additional research is necessary in respect to the impact of demographic characteristics such as sex and education as well as health and cognitive status. No differences in IIV appear to be according to gender (Hultsch et al., 2002; Mella et al., 2016). Conflicting findings exist in respect to the relationship between IIV and educational attainment as well as overall cognitive ability. Hilborn et al. (2009) demonstrated that higher levels of education and overall cognitive ability may be associated with lower levels of IIV. Christensen et al. (1999), Hultsch et al. (2002), and Schretlen et al. (2003) also showed that IIV might decrease with higher ability levels, whereas in the study by Lindenberger and Baltes (1997) the opposite was evident. Such discrepancies in results might be due to methodological issues as both educational attainment and overall cognitive functioning are variables differently operationalized in studies. As an example, to assess overall cognitive ability some researchers use intelligence tests (Lindenberger and Baltes, 1997; Schretlen et al., 2003), whereas others use composite scores of several cognitive measures (Hultsch et al., 2002). Education has also been used a proxy to assess intelligence and overall cognitive ability (Christensen et al., 1999).

In the context of aging and age-related intraindividual differences, exploring EFs seems particularly important. The prefrontal cortex (PFC) is considered the brain region most disrupted by healthy aging (Bryan and Luszcz, 2000; De Luca et al., 2003; Cabeza and Dennis, 2013); therefore, EFs have been proposed as potential mediators of age-related cognitive decline (Levine et al., 1997; Parkin, 1997; Salthouse et al., 2003; Troyer et al., 2007). Indeed, findings support the notion that changes in cognition with advancing aging reflect age-related decline in frontal lobe functioning. For example, evidence exists in respect to word-list-learning performance showing a greater incidence and increased inconsistence in false memory in healthy older adults as compared to younger adults (Murphy et al., 2007). Furthermore, patients with frontal lobe lesions showed greater dispersion and inconsistency in respect to reaction time (RT) tasks performance relative to patients with non-frontal lesions (Stuss et al., 2003). Additionally, increased IIV in RT and episodic memory performance among healthy individuals have been associated with smaller prefrontal white matter volumes (Lövdén et al., 2013) and frontal white-matter hyperintensities (Bunce et al., 2007). On the other hand, studies investigating IIV in EFs that use standardized neuropsychological tests are scarce. In most cases, IIV has been examined using EF tasks in combination with other neuropsychological measures. In particular, verbal fluency and cognitive flexibility measures have been applied (Baltes and Lindenberger, 1997; Lindenberger and Baltes, 1997; Schretlen et al., 2003; Rapp et al., 2005). As findings derived from these studies refer more to cross-domain variability, additional research focusing within-domain variability in EFs is required.

The current study was aimed at exploring IIV in EF performance in healthy adults across a large life span. The focus was on dispersion as this is the aspect of IIV that is not well studied, neither in respect to cognition in general nor in respect to EFs. The main goal was to clarify whether there are age-related changes in across-tasks variability in EF performance by examining cross-sectional associations between dispersion and age. As the most of studies conducted are comprised of older adults, the current research was aimed at exploring dispersion in young participants as well. Furthermore, the goal was to compare different stages of adulthood with regard to the magnitude of dispersion. In addition to age, the impact of educational attainment, and sex was investigated. In line with the previous research (Lindenberger and Baltes, 1997; Hilborn et al., 2009; Heyanka et al., 2013), the current study examined differences in dispersion according to overall cognitive performance. Based on previous findings regarding dispersion in healthy adults (Lindenberger and Baltes, 1997; Rapp et al., 2005) as well as findings showing age-related increases in the intercorrelationship among EFs (Buczylowska and Petermann, 2017), it was hypothesized that across-tasks variability in EF performance would decrease in old age. In line with the previous research (Christensen et al., 1999; Mella et al., 2016), sex was not expected to exert an impact on the dispersion level. Due to conflicting prior findings, no hypotheses were formulated with regard to the impact of educational attainment and overall cognitive ability on dispersion.

## Method

### Sample characteristics

Participants were 444 adults (205 males and 239 females) aged 18–99 (*M* = 59.74; *SD* = 20.35), recruited for the purpose of norming the German adaptation of the Neuropsychological Assessment Battery (NAB; Petermann et al., 2016b). Data were collected on four different sites in Germany, including the north, south, west, and east parts of the country, between February 2014 and February 2015.

To better understand the changes in IIV across the life span, the sample was subdivided into four age groups. The young age group (*N* = 98, 48 men, 50 women) ranged from 18 to 39 years (*M* = 28.69, *SD* = 6.29). The middle age group (*N* = 89, 39 men, 50, women) ranged from 40 to 59 years (*M* = 51.24, *SD* = 5.28). The middle-old age group (*N* = 134, 65 men, 69 women) ranged from 60 to 74 years (M = 67.42, *SD* = 4.26). The old age group (*N* = 123, 53 men, 70 women) ranged from 75 to 99 years (*M* = 82.27, *SD* = 5.35). Sample characteristics in respect to education are presented in Table 1.

**Table 1:** Demographic characteristics of the sample.

	**Education/type of school**, ***N*** **(%)**				
**Age**	**Hauptschule/ Volksschule^a^**	**Realschule^b^**	**Abitur^c^**	**Valid *N***	**Total *N***
18–39 years	21(21.4)	35(35.7)	42(42.9)	98	98
40–59 years	9(10.1)	37(41.6)	43(48.3)	89	89
60–74 years	34(25.6)	70(52.6)	29(21.8)	133	134
75–99 years	58(47.2)	44(35.8)	21(17.1)	123	123
Sample	122(27.5)	186(42)	135(30.5)	443	444

a *8–9 years of mandatory school*.

b *10 years of advanced school*.

c *A-level equivalent after regular 13 years of school*.

Potential participants with known cardiovascular, neurological, or psychiatric conditions were excluded from the sample. Written informed consent was obtained from all participants. Only participants who completed Form 1 of the NAB on the first occasion were included in the study.

### Assessment tools

#### NAB executive functions module

The NAB is a battery of neuropsychological tests designed for the assessment of cognitive functions in adults with disorders of the central nervous system (White and Stern, 2003). The NAB is composed of one screening-module and five domain-specific modules (i.e., Attention, Language, Memory, Spatial, and Executive Functions). Performance on the five domain-specific modules results in the NAB Total Index, which is the standard score for overall cognitive functioning.

Due to standardization procedures, in which all tests are normed on a single standardization sample, the NAB Executive Functions Module offers a set of conormed tasks, suitable for the assessment of various aspects of executive functioning. Additionally, the Executive Functions Index (EFI) is available as a measure of overall performance.

Within the German adaptation of the NAB, the four original subtests of the Executive Functions Module were translated into German and adapted to standard conditions in German-speaking countries (Buczylowska et al., 2013). In the Executive Functions Module of the German NAB adaptation two additional subtests are included: Planning (German *Planen*) and Letter Fluency (German *Wortflüssigkeit*); however, only Letter Fluency offers a standard score and contributes to the EFI and the NAB Total Index. Planning is an adopted task based on the “Bogenhausener Planungstest” (von Cramon, 1988; von Cramon et al., 1991), an experimental measure designed to assess complex planning skills in the context of daily living. Letter Fluency is a task designed by the authors of the German NAB adaptation; it is based on the concept of verbal fluency (Strauss et al., 2006; Lezak et al., 2012). A detailed description of the German NAB Executive Functions Module is presented in Table 2.

**Table 2:** Description of the Executive Functions Module of the German NAB adaptation.

**Subtest**	**Task description**	**Function**
Planning	The examinee is to put 5 typical daily-living time-restricted assignments into the correct order during a fixed period of time of max. 15 min.	Planning, problem solving, implementing strategies, mental flexibility.
Mazes	The examinee is to complete 7 time-restricted, paper-and-pencil mazes of increasing difficulty.	Planning, impulse control, psychomotor speed.
Letter Fluency	The examinee is to create as many words as possible with specified initial letter during 120 s.	Verbal fluency, generativity.
Judgment	The examinee is to answer 10 judgment questions pertaining to daily living issues associated with home safety, health, and medicine.	Problem solving and decisional capacity in daily living situations.
Categories	The examinee is to generate different two-group categories based on photographs and verbal information about six people. The task is composed of two panels, each of 240 s.	Concept formation, cognitive response set, mental flexibility, generativity.
Word Generation	The examinee is to create three-letter words based on a visually presented group of eight letters (three vowels, five consonants) under the time restriction of 120 s.	Verbal fluency, generativity.

For the German NAB Executive Functions Module following reliability coefficients are reported: internal consistency reliability, α = 0.82; test-retest reliability for younger age ranges (18–69 years old), *r* = 0.86, and for older age ranges (70–>85), *r* = 0.85 (Petermann et al., 2016a).

## Analyses

Statistical analyses were performed using Microsoft Office Excel 2007 and IBM SPSS Statistics 24. First, raw data from the Executive Functions Module subtests (i.e., Planning, Mazes, Letter Fluency, Judgment, Categories, and Word Generation) were z-transformed. Maximum discrepancy (Schretlen et al., 2003; Heyanka et al., 2013; MD) was employed as a measure of dispersion; that is, the difference between the highest and lowest scores for each person across the six subtests was calculated. Greater MD scores imply a relatively uneven performance profile, whereas smaller MD scores indicate a flatter, more consistent cognitive profile. To determine the relationship between dispersion and age, a Pearson correlation coefficient between the MD values and age in years was calculated.

As the MD values might have been affected by age, the six subtests scores were regressed on age. The resulted residuals were saved as z-transformed scores und used to compute MD values and a correlation between the MD values and age again. The z-transformed scores were used for further analyses, too.

To estimate the intercorrelationship between the NAB subtests, intercorrelations for the sample and for the four age groups separately were calculated. In the next step, a univariate analysis of variance (ANOVA) was applied to investigate the difference in MD between the four age groups. At the same time, the factor effects of education and sex were analyzed.

The focus of attention was also on the relationship between dispersion and overall cognitive ability. As a measure of cognitive ability, the NAB Total Index score was used. A Pearson correlation between the MD values and the NAB Total Index scores was calculated. Based on the performance on the NAB Total Index, the sample was subdivided in four groups: 1 *SD* above average, 0–1 *SD* above average, 0–1 *SD* below average, and 1 *SD* below average). Correlations between the MD values and age for the four ability groups were computed.

## Results

Descriptive statistics of the NAB Executive Functions Module for the four age groups and sample are presented in Table 3. MD values based on z-transformed raw scores ranged from 0.31 to 4.97 *SDs* (*M* = 2.01, *SD* = 0.69). 8% of participants produced MD values greater than 3 *SDs*, 40% produced MD > 2 *SDs* and 47% produced MD values greater than 1 *SD*. Only 6% of participants produced MD values up to 1 *SD*. There was a negative correlation between MD and age (*r* = −0.21, *p* < 0.01) implying that dispersion in EFs might moderately decrease with advancing age.

**Table 3:** Descriptive statistics for the NAB Executive Functions Module based on raw scores.

**Subtest**	**18–39 years**	**40–59 years**	**60–74 years**	**75–99 years**	**Sample**
Planning	8.69(2.48)	7.80(3.29)	6.43(3.61)	4.48(3.32)	6.66(3.60)
Mazes	20.12(4.89)	15.11(5.36)	10.48(5.76)	5.46(4.40)	12.15(7.44)
Letter Fluency	16.24(5.56)	18.11(6.22)	15.95(5.70)	13.21(5.25)	15.69(5.90)
Judgment	12.89(1.76)	12.76(1.89)	11.97(1.99)	10.61(2.16)	11.95(2.17)
Categories	26.17(9.68)	22.76(8.48)	16.72(8.07)	10.14(6.79)	18.19(10.20)
Word Generation	7.55(2.89)	7.76(2.92)	7.13(2.81)	5.84(2.92)	6.99(2.99)

*Data represent means ± SD*.

MD values based on z-transformed residuals ranged from 0.47 to 5.20. That is, age might have slightly inflated the MD values. This is also reflected in the distribution of the MD values in the sample, so that 15% of participants produced MD values greater than 3 *SDs*, 43% produced MD values greater than 2 *SDs* and 39% produced MD values greater than 1 *SD*. Only 4% of participants produced MD values up to 1 *SD*. Furthermore, 53% of the participants exhibited in one or more subtests a score below 1 *SD* and 8% of the participants exhibited in one or more subtests a score below 2 *SD*s below average. The test scores of participants whose MD values exceeded 3 *SD* were reviewed to determine which subtests most frequently contributed to the highest dispersion levels. Among subtests with the lowest or highest score in all 65 participants with MD values above 3 *SD*, Mazes was involved 29 times, Letter Fluency 28 times, Planning 20 times, Judgment and Categories 19 times, and Word Generation 10 times. However, Planning contributed most frequently to MD values as the lowest score (26%), followed by Mazes and Judgment (20%), Letter Fluency (15%), Categories (11%), and Word Generation (8%). The analysis showed that in 49 individuals (75%) the lowest score exceeded 1 *SD* below average.

The intercorrelations among the six subtests (see Table 4) ranged in the sample from *r* = 0.13 to *r* = 0.42. The mean intercorrelation was *r* = 0.25. Based on the intercorrelations, the shared variance between the subtests ranged from 0.2% to 13% in the 18–39 age group, from 0.01% to 13% in the 40–59 age group, from 0.8% to 14% in the 60–74 age group and from 0.7% to 29% in the 75–99 age group. The correlation between age in years and MD values based on z-transformed residuals (*r* = −0.14, *p* < 0.01) was still significant. A 4 (age) × 2 (sex) × 3 (education level) ANOVA revealed a significant effect of age, [*F*_(3, 442)_ = 3.43, *p* = 0.017, η2 = 0.02] for MD values. The factor effects of gender and education level were not significant. Bonferroni type post-hoc analyses revealed significant differences in MD values (*p* < 0.05) between the old age group (*M* = 1.80) and any other group. That is, the 75–99-year-olds showed less dispersion than the participants of younger age groups. There were no significant differences between the young age group (*M* = 2.19), the middle age group (*M* = 2.08), and the middle-old age group (*M* = 2.03).

**Table 4:** Intercorrelations between the NAB Executive Functions Module subtests.

**Subtest**	**Planning**	**Mazes**	**Letter Fluency**	**Judgment**	**Categories**	**Word Generation**
Planning	–	0.25	0.13	0.22	0.30	0.25
Mazes	0.25	–	0.13	0.13	0.27	0.24
Letter Fluency	0.13	0.13	–	0.21	0.32	0.42
Judgment	0.22	0.13	0.21	–	0.33	0.16
Categories	0.30	0.27	0.32	0.33	–	0.34
Word Generation	0.25	0.24	0.42	0.16	0.34	–

*p < 0.01; two-tailed probability*.

A small but significant correlation between the MD values and Total NAB Index scores (*r* = 0.10, *p* < 0.05) indicates that overall ability level should also be taken into account. Subdividing the Total NAB Index in four different ability levels (i.e., 1 *SD* above average, 0–1 *SD* above average, 0–1 *SD* below average, and 1 *SD* below average) revealed more insight into the relationship between IIV and age. That is, the correlation between MD values and age varied according to ability level, ranging from *r* = −0.06 for the highest ability group (> 1 *SD*) and *r* = −0.05 for the high average group (+1 *SD*) to *r* = −0.25 for the low average group (−1 *SD*) and *r* = −0.30 for the lowest ability group (> −1 *SD*). This implies that IIV might be lower at older ages, particularly when associated with low overall ability levels.

## Discussion

The current study demonstrates considerable dispersion in respect to performance on six NAB EF tasks. This is based on the observation that 96% of the participants produced MD values exceeding at least 1 *SD*. These findings are in line with those derived from previous research on dispersion in respect to neuropsychological test performance (Schretlen et al., 2003; Heyanka et al., 2013). 52% of the participants produced MD values exceeding at least 2 *SDs*. Such discrepancies between test scores must be interpreted in the context of neuropsychological evaluations. Given that presumably healthy people were assessed within the current research, discrepancies between test scores in the normal population might be more common than expected. Thus, in the clinical context especially, more attention should be paid to the distribution of test scores in relation to the MD values found in the population. Furthermore, in the present study, 53% of the participants exhibited in one or more subtests a score below 1 *SD* and 8% of the participants exhibited in one or more subtests a score below 2 *SD*s below average. As scores exceeding 1 *SD* below average are considered an indication of cognitive impairment, lower cutoffs to identify cognitive problems might be more appropriate for clinical use (Brooks et al., 2009).

The current results imply that dispersion in EFs might moderately decrease with advancing age. This is in contrast to previous research since the majority of studies showed an age-related increase in dispersion (Christensen et al., 1999; Hultsch et al., 2002; Schretlen et al., 2003; Stuss et al., 2003; Hilborn et al., 2009; Ferreira et al., 2017). As already suggested by other researchers, inconsistent findings might be due to the age range investigated (Hultsch et al., 2002; Hilborn et al., 2009). If a large life span is examined, there might be increases in dispersion in young and healthy adults followed by decreases in dispersion in very old adults as later in life, cognitive deteriorations are more likely (Hultsch et al., 2002). Additionally, age-related disturbances might often occur within more than one cognitive domain (de Frias et al., 2007); as a result, more consistent cognitive profiles and less task dispersion could frequently be found in very old adults. However, this pertains to deterioration patterns associated with normal aging. As suggested by Rapp et al. (2005) this might be in contrast to deterioration patterns induced by cerebral dysfunctions, which may be associated with specific deficits in only one domain of cognitive functioning. Consequently, increases in across-tasks variability are more likely in adults affected by cerebral dysfunctions than in those with age-appropriate cognitive profiles. This is important since the current study is based on a test norming sample drawn upon strict exclusion criteria. That is, potential participants with known cardiovascular, neurological, or psychiatric conditions were excluded from the participation in the study. Additionally, selective mortality is likely to restrict the range of interindividual variability at the lower end of ability spectrum (Lindenberger and Baltes, 1997). As a result, individuals reaching very old age might be less likely to be diagnosed with clinically relevant diagnoses, and thus, they might also be cognitively healthier. Moreover, in contrast to prior research often using convenience samples, the current study is based on a representative sample of healthy adults. Hence, the current findings might more accurately reflect dispersion trends in the normal population.

The current findings are consistent with three previous studies (Lindenberger and Baltes, 1997; Rapp et al., 2005; Mella et al., 2016). Rapp et al. (2005) reported higher levels of dispersion in nursing home residents than in community-dwelling older adults. In adults living in the community dispersion decreased with advancing age. Rapp et al. (2005) argued that this may reflect the absence of cerebral dysfunction. Lindenberger and Baltes (1997) examined IIV in intellectual abilities at old and very old ages (i.e., 70–102 years) and found decreases in dispersion with age of similar magnitude (i.e., *r* = −0.19) as in the current research. In the current study, a significant difference in dispersion was observed between the oldest group (i.e., 75–99 years) and all three younger groups of the 18–74 age range. Interestingly, no significant differences in dispersion were evident between the younger age groups; thus, it is likely that the shift in the magnitude of dispersion happens relatively late in life. As suggested by other researchers (Lindenberger and Baltes, 1997; Rapp et al., 2005; Mella et al., 2016), decreases in dispersion might reflect dedifferentiation processes, which are primarily dominated by aging-induced changes in brain integrity. The dedifferentiation in cognition that occurs at old age is characterized by the linear decrement of cognitive performance and greater magnitude of intercorrelations between cognitive abilities than that observed at younger ages. The phenomenon of greater uniformity between cognitive abilities in old age has already been demonstrated on an example of increasing correlationship between EFs and intelligence (Buczylowska and Petermann, 2017). Age-related increases in correlations among different cognitive abilities were reported elsewhere, too (Baltes and Lindenberger, 1997; Deary et al., 2004; de Frias et al., 2007). In the present study, the magnitude of intercorrelations between the subtests increased from 0.2–13% in the 18–29 age group to 0.7–29% in the 75–99 age group. Additionally, as already demonstrated in a previous study (Buczylowska and Petermann, 2016), performance on the NAB Executive Functions Module subtests is marked by decrement in all test scores across age. Planning, Mazes and Categories were the subtests with the highest decrease in mean scores and the highest increase in interindividual variability; whereas Letter Fluency, Judgment and Word Generation were the subtests with the lowest decrease in mean scores and the lowest increase in interindividual variability. If there are differences between tasks in deterioration patterns, there might also be differences in intraindividual variability. However, differences between individuals are not necessarily associated with differences within individuals as individuals differing in performance among each other might show consistent performance within each other. Nevertheless, it is meaningful to analyze how the individual NAB subtests contribute to across-tasks dispersion and whether this is associated with the deterioration pattern identified in the previous research. In 65 participants whose MD values exceeded 3 *SD*, Mazes and Letter Fluency were the subtests most frequently involved as the highest or lowest score, followed by Planning, Judgment and Categories, and Word Generation. However, Planning, Mazes, and Judgment contributed most frequently to MD values as the lowest score. As identified in the previous research, Planning and Mazes were also the subtests with the highest increase in interindividual variability. On the one hand, high dispersion levels might be partly due to tasks more frequently resulting below average scores. On the other hand, the various characteristics of the individual tasks might be responsible for uneven performance profiles, too. Based on the various underlying skills, the NAB subtests can be differently classified. Planning and Mazes are paper-pencil time restricted tasks. In particular, Mazes involves visuo-spatial skills and speed. This is in contrast to the other NAB subtests, in particular highly language-related Letter Fluency and Word Generation. Consequently, different skills involved must be taken into account when considering across-tasks dispersion levels.

Inconsistent findings with regard to IIV in cognitive performance might also be due to various domains investigated and assessment tools used. In the previous research, RT tasks and various neuropsychological measures were frequently used. In contrast, the current study focused solely on the assessment of EFs and used a set of conormed tasks. Mella et al. (2016) examined across-tasks dispersion in children, young adults, and older adults separately for RT and WM tasks. Dispersion in RT tasks was characterized by a U-shaped curve with heterogeneous performance both in children and older adults. With regard to WM tasks on the other hand, young adults displayed greater dispersion than children and older adults. Thus, as suggested by Mella et al. (2016), dispersion across RT tasks and dispersion across WM tasks might be driven by different processes. Moreover, age-related cognitive impairments may be more likely to occur in more than one aspect of a cognitive domain. Consequently, there might be less task dispersion in adults with age-related executive disfunctions.

In line with the expectations, there was no effect of sex on the level of dispersion. As demonstrated by previous studies (Lindenberger and Baltes, 1997; Christensen et al., 1999; Hultsch et al., 2002; Schretlen et al., 2003; Hilborn et al., 2009) educational attainment and the level of overall ability might be linked to IIV in cognitive performance. Contrary to the previous results, dispersion in the current study was independent of educational attainment. It must be noted that years of schooling may not be an appropriate measure of educational attainment. Due to cohort effects, there might be discrepancies between participants of different ages with the same number of years of schooling. Moreover, educational attainment is likely to be related to the level of overall cognitive ability. The current research implies that dispersion may be positively related to overall cognitive ability. Moreover, taking overall ability into account might also reveal more insight into the relationship pattern between age and dispersion; that is, the negative relationship between age and dispersion appears to be stronger in adults with low overall ability levels. Lindenberger and Baltes (1997) obtained similar findings by demonstrating a negative relationship between across-tasks dispersion and age in low ability participants. They argued that very old, low ability participants have the most dedifferentiated pattern of cognitive performance as they are likely to perform uniformly low across all tasks. The flattering of the cognitive profile in low ability adults may be explained by central nervous system constraints associated with very old age. The dedifferentiation hypothesis appears to be a plausible explanation for the findings from the current study, too. Decreases in dispersion with advancing age and lower overall cognitive functioning as well as increasing intercorrelations between tasks demonstrate that dedifferentiation processes may apply to healthy, community-dwelling older adults.

## Limitations

There are several potential limitations to the current study. First, due to cross-sectional study design, it must be noted that observed differences in dispersion do not reflect developmental changes but differences between age groups. Cohort effects may apply to educational attainment, health, life style, and overall cognitive ability. Longitudinal studies are considered more informative in respect to cognitive changes over time. Especially in respect to changes in dispersion longitudinal studies are preferred as they are based on within-person comparisons between different occasions.

Second, the decision to divide the sample into four age groups might have influenced the study results. That is, when examining the adult life span, comparing dispersion levels between different age groups might be affected by the age range of the individual age groups. In particular, in larger age groups comprised of individuals with different levels of overall cognitive ability, only general conclusions regarding age-related differences in dispersion are allowed. Thus, when using a cross-sectional study design, studies with several small age groups are recommended.

Third, the NAB Executive Functions Module scores were used as a measure of EFs and also, as a part to the Total NAB Index, contributed to the measure of overall ability. Consequently, the Total NAB Index cannot be considered as an independent measure of overall ability level. That is, when exploring the relationship between the Total NAB Index and any other NAB module, it must be taken into account that the module scores contribute twice to the analysis.

Fourth, due to the lack of research on IIV in EFs, the results of the present study were discussed mainly in relation to the available findings on IIV in different cognitive domains. As there might be differences in IIV according to cognitive domain, additional research targeting executive functioning in its various aspects and using different assessments tools will be required. Consequently, the nature of the tasks used should be taken into account. Although the NAB offers a comprehensive assessment of executive functioning, there might be executive aspects more pronounced, whereas some other aspects may be neglected. Furthermore, the NAB Executive Functions Module includes complex, multifaceted tasks that are appropriate for ecologically valid use in the clinical practice. Due to practical implications it is meaningful to investigate IIV in EFs measured by such assessment tools. Nevertheless, future studies should also focus on basic aspects of executive functioning, such as updating, shifting, and inhibition as proposed by Miyake et al. (2000), especially because these executive components are among those frequently examined in respect to other research questions.

## Conclusion

To conclude, the current study demonstrates considerable across-tasks dispersion in respect to EF performance. When taking age into account dispersion appears to decrease with advancing age and reach its lowest level late in life. Furthermore, lower levels of overall cognitive ability may be associated with decreases in dispersion, too. The current findings can be accounted for by the representativeness of the sample, the absence of cerebral dysfunctions in participants, and dedifferentiation processes associated with normal aging. These findings should be considered in the context of clinical evaluations, especially because lower cutoffs to identify cognitive problems might be more appropriate for clinical use. Additionally, assessment tools designed to detect dispersion as well as base rates of dispersion for different ages could be useful to identify cognitive impairments associated with pathological aging.

## Ethics statement

This study was carried out in accordance with the recommendations of Ethical Principles of Psychologists and Code of Conduct of the American Psychological Associations and Ethical Pronciplies of the Swiss Psychological Association, Ethical committee of the Philosophy Faculty of the University of Zürich with written informed consent from all subjects. All subjects gave written informed consent in accordance with the Declaration of Helsinki. The protocol was approved by the Ethical committee of the Philosophy Faculty of the University of Zürich.

## Author contributions

DB conceived the study and was involved in data collection. DB also performed statistical analysis and drafted the manuscript. FP supervised the study and provided feedback to the draft of the manuscript.

### Conflict of interest statement

FP is the editor of the German NAB adaptation. The other author declares that the research was conducted in the absence of any commercial or financial relationships that could be construed as a potential conflict of interest.
